# Transcriptomic profiles of human foreskin fibroblast cells in response to orf virus

**DOI:** 10.18632/oncotarget.17417

**Published:** 2017-04-25

**Authors:** Daxiang Chen, Mingjian Long, Bin Xiao, Yufeng xiong, Huiqin Chen, Yu Chen, Zhenzhan Kuang, Ming Li, Yingsong Wu, Daniel L. Rock, Daoyuan Gong, Yong Wang, Haijian He, Fang Liu, Shuhong Luo, Wenbo Hao

**Affiliations:** ^1^ Institute of Antibody Engineering, School of Laboratory Medicine and Biotechnology, Southern Medical University, Guangzhou, 510515, P.R. China; ^2^ Department of Laboratory Medicine, Guangzhou General Hospital of Guangzhou Military Command of PLA, Guangzhou, 510010, P.R. China; ^3^ Guangdong Provincial Key Laboratory of Tropical Disease Research, School of Public Health, Southern Medical University, Guangzhou, 510515, P.R. China; ^4^ Department of Pathobiology, College of Veterinary Medicine, University of Illinois at Champaign-Urbana, Urbana, IL 61802 USA; ^5^ Department of Laboratory Medicine, School of Stomatology and Medicine, Foshan University, Chancheng District, Foshan, Guangdong Province, 528000 P.R. China; ^6^ Department of Pathophysiology, School of Stomatology and Medicine, Foshan University, Chancheng District, Foshan, Guangdong Province, 528000 P.R. China

**Keywords:** orf virus, transcriptomic profiles, apoptosis, antiviral immune response, cell cycle

## Abstract

Orf virus has been utilized as a safe and efficient viral vector against not only diverse infectious diseases, but also against tumors. However, the nature of the genes triggered by the vector in human cells is poorly characterized. Using RNA sequencing technology, we compared specific changes in the transcriptomic profiles in human foreskin fibroblast cells following infection by the orf virus. The results indicated that orf virus upregulates or downregulates expression of a variety of genes, including genes involved in antiviral immune response, apoptosis, cell cycle and a series of signaling pathways, such as the IFN and p53-signaling pathways. The orf virus stimulates or inhibits immune gene expression such as chemokines, chemokine receptors, cytokines, cytokine receptors, and molecules involved in antigen uptake and processing after infection. Expression of pro-apoptotic genes increased at 8 hours post-infection. The p53 signaling pathway was activated to induce apoptosis at the same time. However, the cell cycle program was promoted after infection, which may be due to the immunomodulatory genes of the orf virus. This presents the first description of transcription profile changes in human foreskin fibroblast cells after orf virus infection and provides an in-depth analysis of the interaction between the host and orf virus. These data offer new insights into the understanding of the mechanisms of infection by orf virus and identify potential targets for future studies.

## INTRODUCTION

Orf virus (ORFV), a type strain of parapoxvirus, causes contagious ecthyma, characterized by inflammation of the wounded epithelial tissue. Its genome consists of linear double-stranded DNA approximately 138 kbp long and encoding 132 genes [1]. Essential genes involved in viral replication, morphogenesis of mature virions and extracellular virions are located in the conserved central part of the genome [2]. Variable genes are in the terminal ends, encoding factors with important roles in virulence, pathogenesis, immune evasion/modulation and host range [1, 2].

ORFV is the causative agent of contagious ecthyma, a non-systemic and contagious acute skin disease that has a worldwide distribution and brings serious economic loss to farmers. It affects not only ruminants, but also people who are in close contact with infected livestock [3–5]. In contrast to orthopoxviruses, human ORFV infections are typically self-limiting and recover completely with routine wound care and antibiotic agents [3, 4, 6]. ORFV has strong immunomodulatory activities [7]. It can cause repeated infections, even in the presence of antibodies. These properties make ORFV an ideal viral vector for disease therapy. Pre-clinical trials using ORFV identified its anti-tumoural activity via activation of both cytokine-secreting and tumoricidal natural killer (NK) cells [8]. Another study stated that perioperative therapy with ORFV could enhance the function of NK cells, which reduces metastatic recurrence in surgical animal models bearing cancer cells [9]. ORFV may also be developed as an immunomodulation application to treat chronic viral infections, liver fibrosis/cirrhosis and immune disorders [7].

While there is an interest in the use of ORFV as an immunomodulation therapy for pathogens, tumors and other diseases, little is known of the impact of ORFV on the human expression profile. Next generation sequencing technology allows monitoring of the expression of host genes in response to infection, which provides new insights into host gene expression, regulation and interactions between the virus and host.

In this study, we carried out the analysis of transcriptome changes in human foreskin fibroblast cells (HFF-1) following ORFV infection via RNA-seq. The results provide a better understanding of the mechanisms of orf virus infection and will aid in the development of ORFV immunomodulation therapies against pathogens, tumors, liver fibrosis/cirrhosis and immune disorders.

## RESULTS

### Distinct and dynamic changes in the expression of cellular genes in ORFV-infected HFF-1 cells

**Figure 1:**
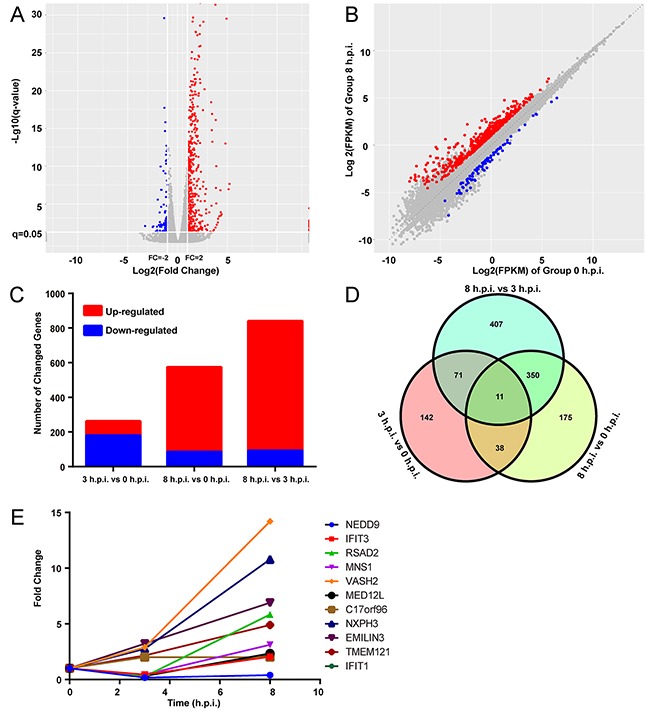
A number of differentially expressed genes were induced by ORFV **(A)** Volcano plot for the samples 8 hours post infection with mRNA expression differences, compared to hour 0. Log2(fold change) is plotted as the abscissa and log10(q) is plotted as the ordinate. Significantly up-regulated genes are indicated in red and down-regulated genes are indicated in blue. **(B)** Scatter plot of the the 0 h.p.i and 8 h.p.i groups is shown. **(C)** Graph showing the number and fold change of up- and down-regulated genes. **(D)** A Venn diagram presents the number of differentially expressed genes that are unique or shared in every paired group. **(E)** The expression patterns of 11 genes that were shared in the three paired groups in (D). q= FDR corrected P-values, FPKM= fragments per kilobase of exon model per million mapped reads.

The transcriptome data was generated from the mRNA isolated from HFF-1 cells cultured with ORFV (MOI=5) at 0, 3 and 8 h post-infection. Each of the data sets contained between 17.8–23.5 million reads and a mapping rate of 97-98% (Table 1). The sample 0h-1 was discarded, because of a clear separation between it and the other two samples, by PCA (Supplementary Figure 1A). The representative distributions of genes up- or down-regulated are shown in the volcano and scatter plots at 8 h post-infection compared to uninfected cells (Figure 1A, 1B). The red dots represent the up-regulated genes, while the blue dots represent the down-regulated genes. At 3 and 8 h.p.i, 82 and 489 genes were up-regulated, respectively, while 180 and 85 genes were down-regulated, respectively (Table 1 and Figure 1C). Compared to the 3 h.p.i. group, 91 genes were down-regulated and 748 genes up-regulated at 8 h post-infection (Figure 1C). From the time course, the expression levels of the majority of genes descended before rising. Compared to the uninfected sample, the 3 h.p.i. and 8 h.p.i. groups shared 49 differentially expressed genes. Among them, 25 genes were commonly up-regulated, 19 were commonly down-regulated, and 5 declined before ascending (Supplementary Figure 1B). Representatives of expression pattern for commonly up-regulated and down-regulated genes are shown in Supplementary Figure 1C and 1D. Most of the up-regulated genes showed the same pattern of being highly up-regulated at 8 hours after infection (Supplementary Figure 1C), while obviously down-regulated genes occurred mainly at 3 hour post-infection (Supplementary Figure 1D).

**Table 1:** Summary of the number of reads mapping for each transcriptone data set and the number of genes significantly up- or down-regulated (normalized to uninfected samples)

Samples_ID	Accession no.	All reads	Mapped reads	Mapping ratio	Number of up-regulated genes	Number of down-regulated genes
0h_1	GSM2448850	19,763,619	19,354,510	97.93%	_	_
0h_2	GSM2448851	19,833,603	19,425,357	97.94%		
0h_3	GSM2448852	21,917,869	21,471,619	97.96%		
3h_1	GSM2448853	17,813,942	17,421,247	97.80%	82	180
3h_2	GSM2448854	18,344,341	17,951,313	97.86%		
3h_3	GSM2448855	21,107,755	20,653,563	97.85%		
8h_1	GSM2448856	23,514,308	22,907,363	97.42%	489	85
8h_2	GSM2448857	20,416,737	19,884,667	97.39%		
8h_3	GSM2448858	23,134,104	22,489,211	97.21%		

In-depth analyses using the data from the three paired groups revealed that a total of 1194 DEGs were regulated by ORFV. The Venn diagram displayed the differently and similarly regulated genes between each two groups (Figure 1D). Meanwhile, the three paired groups shared 11 genes that were differentially expressed (Figure 1E). The expression patterns of three interferon-inducible genes (*IFIT3*, *IFIT1* and *RSAD2)* were decreased before beginning to increase. The expression level of *VASH2*, a gene encoding angiogenesis inhibitor, was sharply elevated. These results indicate that ORFV can increase and decrease expression levels of a number of host genes.

### Gene ontology analysis of the differential genes

**Figure 2:**
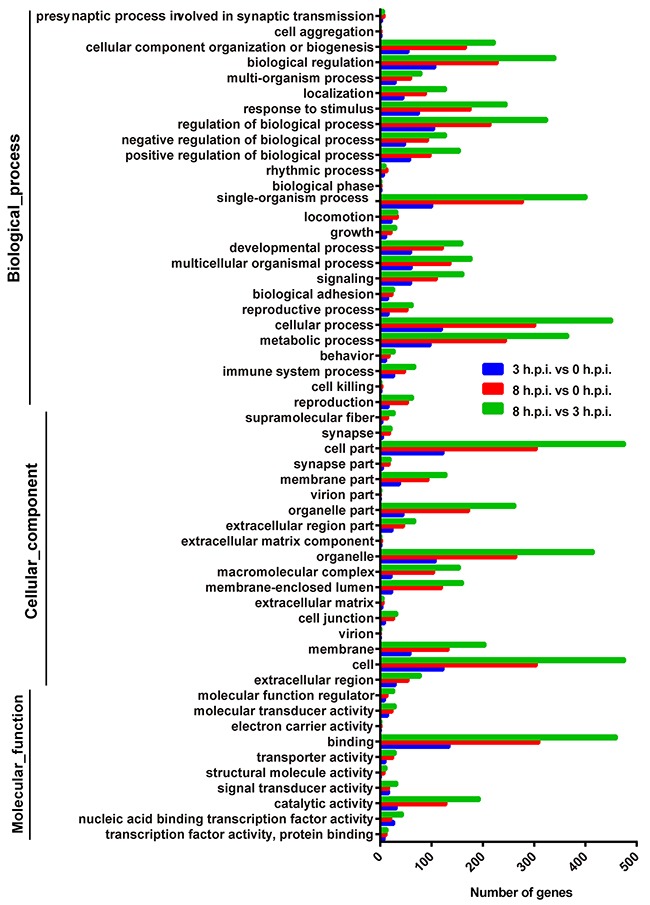
Functional analyses of the differentially expressed genes by Gene Ontology (GO) classifications for the three paired groups The abscissa represents the enriched gene number. Blue = group 3 h.p.i. vs. group 0 h.p.i, red = group 8 h.p.i. vs. group 3 h.p.i, green = group 8 h.p.i. vs. group 3 h.p.i.

In order to gain a better understanding of the associated functions for the differentially expressed genes, gene ontology (GO) analysis was used to perform an enrichment analysis and classifications (Figure 2). GO analysis identified enriched biological processes associated with “cellular component organization or biogenesis”, “biological regulation”, “response to stimulus”, “regulation of biological process”, “single-organism process”, “cellular process” and “metabolic process”, indicating that strong host response were induced by the virus. Identified enriched cellular component terms associated with “cell part”, “organelle part”, “organelle”, “membrane” and “cell”, suggesting multifarious cellular components involved in the viral infection and replication. Enriched molecular functions were defined associated with “binding” and “catalytic activity”, implying that specific interactions between host and virus were occurring.

**Figure 3:**
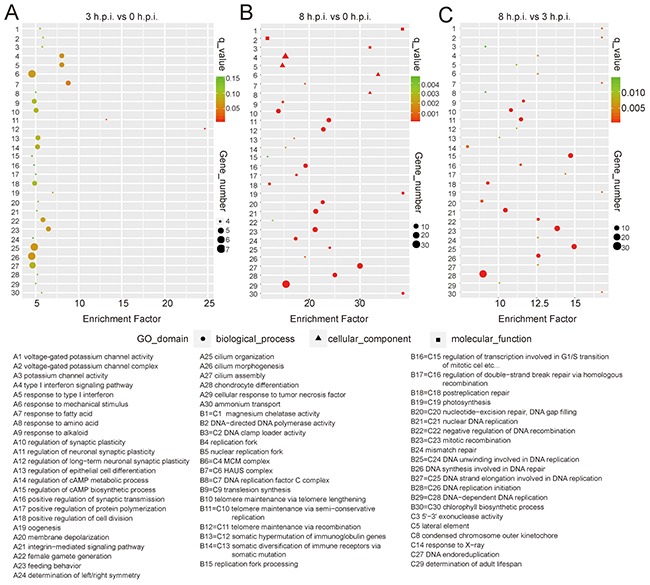
Function enrichment of the DEGs from the transcriptome data by GO analysis The figure presents the GO enrichment of the samples at 3 h.p.i. **(A)** or 8 h.p.i. **(B)**, compared to uninfected cells. **(C)** GO enrichment is shown for group 8 h.p.i. against group 3 h.p.i. Enrichment factor is plotted on the x axis. The size of each bubble represents the number of genes.

Compared to uninfected samples, GO analysis showed that the type I interferon signaling pathway was significantly enriched at 3 h.p.i. (p<0.05). Most enriched genes in the pathway were down-regulated at 3 h.p.i. but were upregulated at 8 h.p.i. (Figure 3A, Supplementary Table 1), inferring that an immunosuppressive effect was induced by the orf virus at early stages, while a potential defend in the cells emerged to block viral replication at late stages. Genes with GO terms associated with neuronal synaptic plasticity and fatty acid had significantly changed at 3 h post-infection (p<0.001) (Figure 3A). At 8 h post-infection, genes with GO terms associated with DNA replication and repair were significantly enriched (Figure 3B and 3C), suggesting that virus was replicating rapidly.

### Analysis of important KEGG pathways

**Figure 4:**
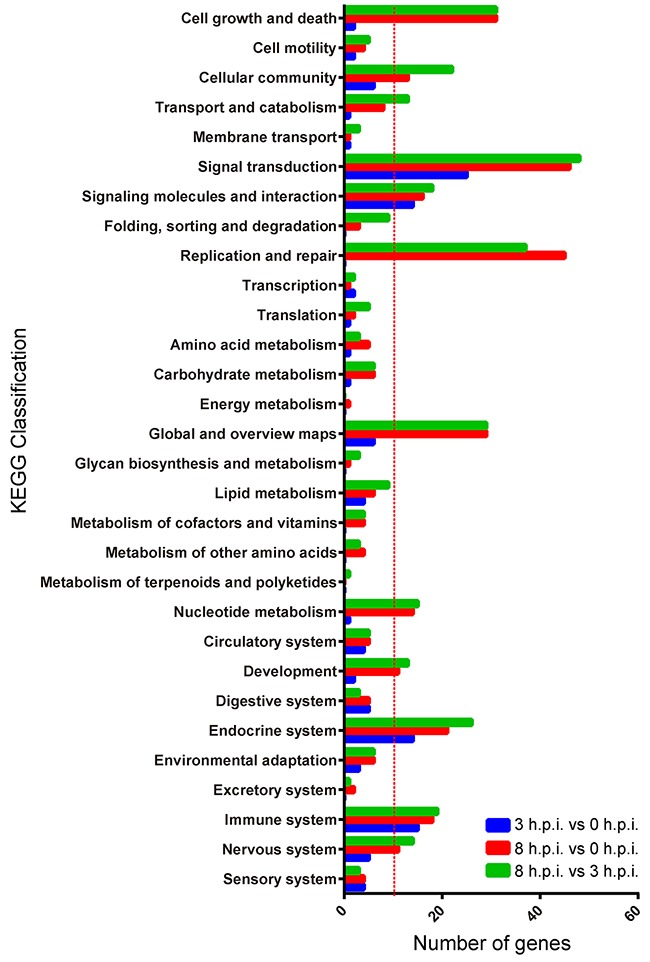
KEGG classifications of differentially expressed genes for the three paired groups Blue = 3 h.p.i. group vs. 0 h.p.i. group, red = 8 h.p.i. group vs. 0 h.p.i. group, green = 8 h.p.i. group vs. 3 h.p.i. group.

We used the differential genes in every pair group for KEGG pathway enrichment. The DEGs were significantly enriched in the classifications of “cell growth and death”, “signal transduction”, and “replication and repair” (Figure 4), showing that viral infection and host response were occurring. The classification of “cellular community”, “signaling molecules and interaction”, “global and overview maps”, “nucleotide metabolism”, “development”, “endocrine system”, “immune response” and “nervous response” were also enriched (Figure 4).

The significant KEGG pathways between the 0 h.p.i. and the 3 h.p.i. groups are shown in Table 2. The pathways associated with viral infection (Herpes simplex infection, influenza A, and hepatitis C) and host immune response (Cytokine-cytokine receptor interaction, toll-like receptor signaling pathway, TNF signaling pathway, and intestinal immune network for IgA production) were enriched. At 8 h post-infection, the genes were most enriched in the DNA replication, cell cycle, and other nucleotide metabolism (mismatch repair, homologous recombination, nucleotide excision repair, base excision repair, purine metabolism, pyrimidine metabolism, oocyte meiosis, folate biosynthesis, and one carbon pool by folate) pathways, indicating that the viral infection had elicited a strong host response (Table 3 and 4). At the same time, the p53 signaling pathway was activated to induce apoptosis and regulate the cell cycle (p<0.01).

**Table 2:** KEGG analysis of DEGs between 3 h post infection and uninfected sample groups

pathway_ID	PATHWAY_DES	P value
hsa05168	Herpes simplex infection	1.05E-03
hsa04060	Cytokine-cytokine receptor interaction	2.65E-03
hsa05164	Influenza A	3.44E-03
hsa05310	Asthma	6.02E-03
hsa04933	AGE-RAGE signaling pathway in diabetic complications	6.08E-03
hsa04620	Toll-like receptor signaling pathway	7.44E-03
hsa04668	TNF signaling pathway	8.67E-03
hsa04270	Vascular smooth muscle contraction	1.24E-02
hsa05202	Transcriptional misregulation in cancer	1.60E-02
hsa05160	Hepatitis C	1.87E-02
hsa00600	Sphingolipid metabolism	2.05E-02
hsa04672	Intestinal immune network for IgA production	2.05E-02
hsa05144	Malaria	2.29E-02
hsa05030	Cocaine addiction	2.29E-02
hsa04024	cAMP signaling pathway	2.56E-02
hsa04713	Circadian entrainment	2.63E-02
hsa05221	Acute myeloid leukemia	3.41E-02
hsa04921	Oxytocin signaling pathway	3.63E-02
hsa04924	Renin secretion	4.60E-02

**Table 3:** KEGG analysis of DEGs between 8 h post infection and uninfected sample groups

pathway_ID	PATHWAY_DES	P value
hsa03030	DNA replication	6.02E-22
hsa03460	Fanconi anemia pathway	6.12E-14
hsa03430	Mismatch repair	7.51E-12
hsa04110	Cell cycle	1.29E-09
hsa03440	Homologous recombination	1.56E-09
hsa03420	Nucleotide excision repair	1.63E-07
hsa03410	Base excision repair	7.99E-07
hsa04115	p53 signaling pathway	2.55E-04
hsa05161	Hepatitis B	4.38E-04
hsa00230	Purine metabolism	7.48E-04
hsa00240	Pyrimidine metabolism	1.30E-03
hsa05162	Measles	7.84E-03
hsa04114	Oocyte meiosis	1.16E-02
hsa00790	Folate biosynthesis	1.27E-02
hsa05222	Small cell lung cancer	1.77E-02
hsa05215	Prostate cancer	2.10E-02
hsa00120	Primary bile acid biosynthesis	2.14E-02
hsa00670	One carbon pool by folate	3.28E-02
hsa04916	Melanogenesis	3.71E-02
hsa05206	MicroRNAs in cancer	4.40E-02
hsa04390	Hippo signaling pathway	4.74E-02
hsa05219	Bladder cancer	4.84E-02

**Table 4:** KEGG analysis of DEGs between 8 h post-infection and 3 h post-infection groups

pathway_ID	PATHWAY_DES	P value
hsa03030	DNA replication	4.67E-11
hsa03460	Fanconi anemia pathway	7.15E-10
hsa03440	Homologous recombination	7.57E-09
hsa04110	Cell cycle	7.79E-08
hsa03430	Mismatch repair	1.46E-07
hsa03410	Base excision repair	1.92E-04
hsa00230	Purine metabolism	1.14E-03
hsa03420	Nucleotide excision repair	1.38E-03
hsa03450	Non-homologous end-joining	1.57E-03
hsa00790	Folate biosynthesis	2.06E-03
hsa05162	Measles	2.78E-03
hsa04115	p53 signaling pathway	3.34E-03
hsa00240	Pyrimidine metabolism	4.05E-03
hsa05168	Herpes simplex infection	5.15E-03
hsa05210	Colorectal cancer	7.28E-03
hsa05206	MicroRNAs in cancer	7.40E-03
hsa00670	One carbon pool by folate	7.40E-03
hsa04114	Oocyte meiosis	1.07E-02
hsa05222	Small cell lung cancer	1.23E-02
hsa05161	Hepatitis B	1.41E-02
hsa05217	Basal cell carcinoma	1.60E-02
hsa00062	Fatty acid elongation	1.60E-02
hsa05200	Pathways in cancer	1.75E-02
hsa04550	Signaling pathways regulating pluripotency of stem cells	2.98E-02
hsa04623	Cytosolic DNA-sensing pathway	3.14E-02
hsa05215	Prostate cancer	4.48E-02
hsa04622	RIG-I-like receptor signaling pathway	4.62E-02
hsa05164	Influenza A	4.63E-02
hsa04390	Hippo signaling pathway	4.84E-02
hsa05160	Hepatitis C	5.00E-02

### RNA sequencing revealed divergent gene expression patterns after infection

**Figure 5:**
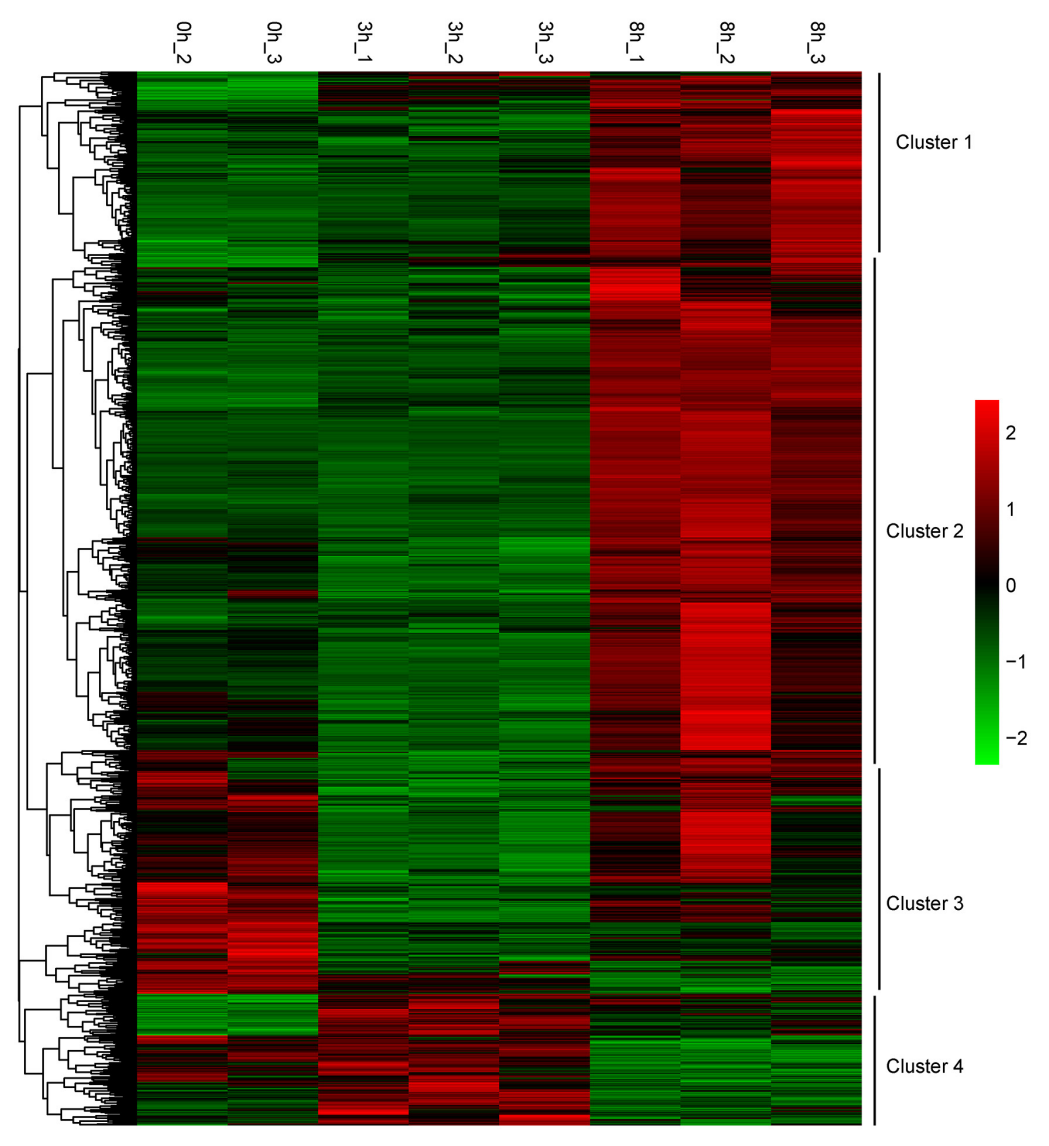
Transcription heatmap of 1194 DEGs for the three groups, with subcluster classification Color scale is shown in the right side. Up-regulated or down-regulated genes are presented by red or green bars.

A hierarchical clustering was performed to generate a heatmap of the 1194 DEGs after stimulation using the FPKM values (Figure 5). These DEGs were partitioned mainly into four distinct expression patterns. Cluster 1 showed a steady increase and cluster 2 changed smoothly before rising. The expression level of cluster 3 was first decreased, and cluster 4 first rose before failing.

**Figure 6:**
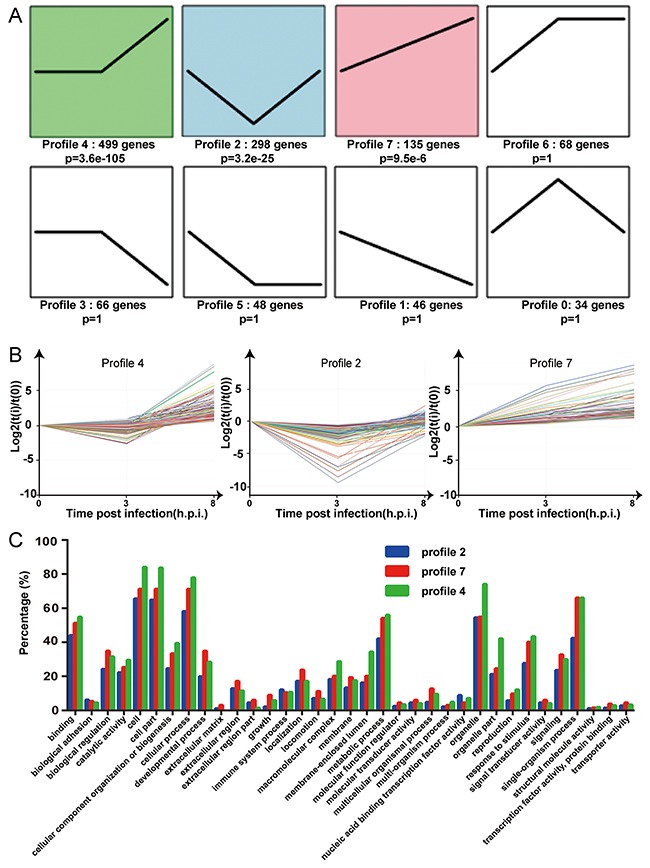
Trend analysis of the 1194 differentially expressed genes **(A)** Different expression patterns of 1194 DEGs were partitioned into 8 profiles. The number of the DEGs and p value assigned to each profile is shown. **(B)** Characteristic expression patterns represented in profiles 2, 4 and 7 (p<0.001). Experimental points are indicated on the x axis: 0 for the uninfected group, 3 for 3 h.p.i, and 8 for 8 h.p.i. The y axis shows the normalized expression values. **(C)** The percentages of the differentially expressed genes related to the GO classifications for the three paired groups are shown. Blue = profile 2, green = profile 4, red = profile 7.

In order to determine the elaborate expression patterns of these differential genes, trend analysis was performed using the OmicShare tools. A total of 8 gene cluster modules were generated for the samples and three profiles (profile 4, 2 and 7) were significantly enriched (P-value <0.001) (Figure 6A). Profile 4 included the largest number of genes (499 genes), indicating that most genes expressed with the model of changing smoothly before rising (Figure 6A and 6B). 298 genes were first decreased (profile 2) and 135 genes were expressed with a steady increase (profile 7) (Figure 6A and 6B). The profiles presented different genetic functions by gene ontology (Figure 6C) and most genes responsible for “binding”, “cell”, “cell part”, “cellular process”, “metabolic process”, “organelle”, “response to stimulus” and “single-organism process”. Profile 4 had a relatively higher percentage of genes responsible for “cell”, “cell part”, “organelle” and “organelle part” than the other two profiles. Profile 4 and 7 had similar percentages of genes responsible for “binding”, “biological regulation”, “cellular process”, “metabolic process”, “response to stimulus” and “single-organism process”, and both of these profiles had enrichment percentages higher than profile 2.

### The DEGs associated with “cell growth and death” and “immune response” induced by orf virus

**Figure 7:**
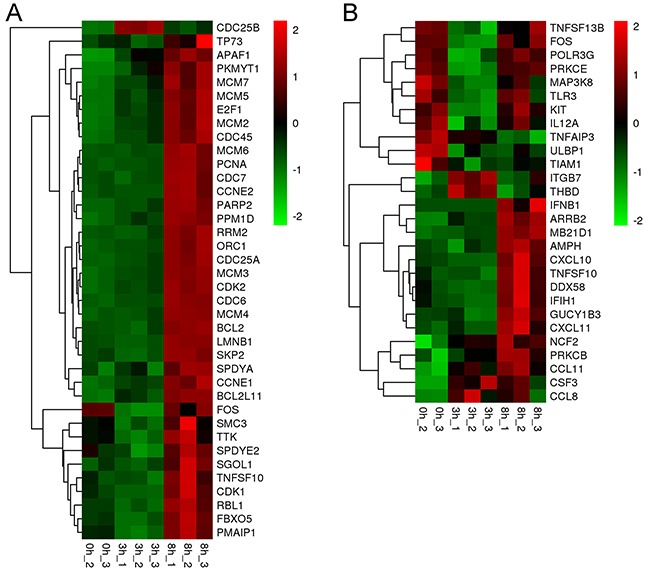
ORFV induced a range of differentially expressed genes associated with “growth and death” and “immune response” **(A)** Significantly (fold change>2 or <-2 and corrected p-value<0.05) up-regulated (red) or down-regulated (green) genes for the different pathways associated to growth and death are shown. **(B)** Significantly up-regulated or down-regulated genes associated with immune response are shown.

Cell growth and death are key processes utilized by the host to regulate lifecycle. The expression of 38 genes associated with cell growth and death were significantly up-regulated by ORFV infection at 8 h post-infection, except for *CDC25B* and *FOS* (Figure 7A and Supplementary Table 2). Among them, many genes are associated with the cell cycle. For example, *E2F1*, *SKP2*, *CCNE1* and *CDC25A* are involved in G1-S transition of the cell cycle. *MCMs* are the putative replicative helicases essential for DNA replication initiation and elongation in eukaryotic cells. *CDC6* is involved in the initiation of DNA replication and participates in checkpoint controls that ensure DNA replication is complete prior to initiation of mitosis. Supplementary Table 3 shows the expression levels of the DEGs involved in the cell cycle. A range of genes that are involved in regulating the cell cycle have been up-regulated, including *CDK1*, *CDK2*, *CDC6*, *E2F1*, *SKP2*, *CCNE1*, *KNTC1*, *BRCA2*, *BCL2* and *CDK5R1*. Of course, negative regulation was also activated. For instance, *RBL1* and *BRCA1*, which participate in negative regulation of the cell cycle, were up-regulated. These data suggest that the cell cycle progression was induced by viral infection. Meanwhile, the expression levels of some genes associated with cell death were significantly increased at 8 h after infection, including *APAF1*, *BCL-2*, *FOS*, *TNFSF10*, *PMAIP1*, *LMNB1*, *PARP2* and *BCL2L11*. For instance, pro-apoptotic gene *BCL2L11* was elevated with an average fold change of 3.51 (corrected p-value <0.001). Eight genes (*APAF1*, *RRM2*, *TP73*, *CCNE1*, *CCNE2*, *CDK1*, *CDK2*, *PPM1D* and *PMAIP1*) associated with the p53 signaling pathway, which can regulate the growth and death of the host cells, were activated. This result indicated that cell growth and death were activated by the orf virus at 8 hours post infection.

Meanwhile, significant changes in gene expression of the DEGs associated with immune response were observed (Figure 7B). The immune function of each gene is listed in Supplementary Table 4. Many genes were down-regulated at 3 hours post-infection, while some were increased. For example, *TNFSF13B*, *FOS*, *POLR3G* and *PRKCE* were down-regulated at 3 h post infection, while *ITGB7* and *THBD* were up-regulated. Most immune genes were up-regulated during the period 3-8 hours post-infection. For instance, *TNFSF13B*, involving in the stimulation of B- and T-cells and the regulation of humoral immunity, was increased 2.13 fold during the time. *TNFAIP3*, *ULBP1* and *TIAM1* were consistently down-regulated by viral infection, while *CCL8*, *CCL11*, *CXCL11*, *NCF2*, *CSF3* and *PRKCB* were significantly up-regulated after infection. Innate immune response and adaptive immunity were activated by these genes. Chemokines were up-regulated to attract T cells, monocytes and granulocytes. These data show that the defense response of host was established in response to viral infection.

### RT-PCR analysis

**Figure 8:**
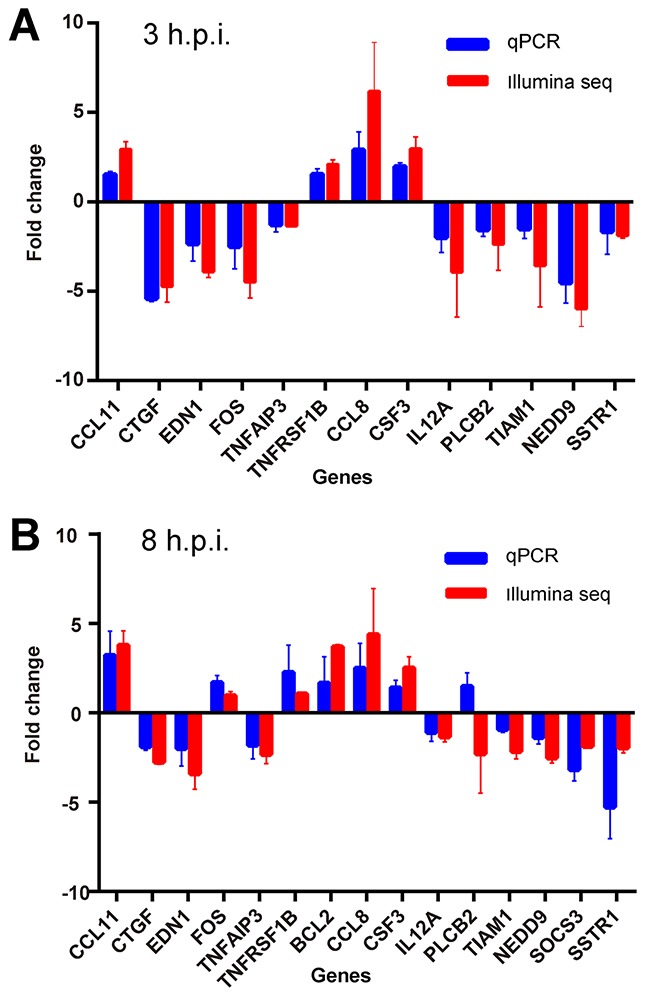
Validation of differentially expressed genes by real-time PCR (RT-PCR) **(A)** RT-PCR was performed with the same samples used in RNA-SEQ. The average value and standard deviation of the independent experiments for the paired group 0 h.p.i vs. 3 h.p.i are plotted in comparison with the fold increase data obtained from Illumina sequencing. **(B)** The results of the paired group 0 h.p.i. vs. 8 h.p.i. are shown.

Fifteen detectable genes from Illumina sequencing were randomly chosen to be further verified by quantitative real-time PCR with the same samples used in RNA-SEQ. Up- or down-regulation of the average RT-PCR fold change for the samples were correlated with that in the sequencing results (Figure 8). Only the expression of *PLCB2* between the two methods was inconsistent. Although some variations were detected, > 90% of the results were reliable. Therefore, the expression changes of the DEGs indeed existed after orf virus infection.

## DISCUSSION

In order to understand the intricate interactions between the host and the orf virus, deep sequencing was performed on mRNA libraries of HFF-1 cells incubated with ORFV. Because the virus can replicate and propagate in HFF-1 cells (Supplementary Figure 2A, 2B), we examined the samples at 0, 3 and 8 h.p.i. with ORFV (MOI=5) according to the cytopathic effects (CPE) in cells incubated with serial dilutions of virus at different time phases (Supplementary Figure 2C, 2D). Nine data sets were obtained from the Illumina platform and contained between 17.4 and 22.9 million reads after trimming and removal of low quality reads. When the data was normalized to the uninfected samples, most down-regulated transcripts could be detected at 3 h.p.i. and were seen to be over-expressed at 8 h.p.i. The expression levels of some genes obtained from qPCR were similar to the data of sequencing, suggesting that the expression profiles from RNA-seq were credible. Some variation existed, as expected, due to the concordance of the two methods [10].

Friebe et al. used microarrays to compare the effect of ORFV and active recombinant stimulation on gene expression patterns in mouse peritoneal cells [11]. Their results indicated that different fragments of ORFV induce/inhibit the transcription of similar genes relevant to the protective effects of ORFV. *CCL3*, *IL15_1*, *CASP11_1*, *RASA4*, and *CCL5* were greatly up-regulated by ORFV NZ2 and ORFV D1701, while *TGFB2*, *APRIL*, *ICAM2* and *ITGA6* were strongly down-regulated [11]. However, the genes identified by Friebe et al. exhibited insignificant expression changes in our experimental system. This discrepancy is likely due to the different cell types utilized in the studies. In the present study, 28 immune genes relevant to the protective effects were detected with significant changes during infection. The genes in the toll-like receptor signaling pathway and TNF signaling pathway were inhibited at early stage. Fleming et al. reported that the inflammation response was limited by several ORFV genes which suppress the production of inflammatory factors [12]. For example, the IL-10-like virokine encoded by *ORFV127* inhibits the production of proinflammatory cytokines, including TNF-α, IL-8, IFN-γ and GM-CSF, in host cells [13]. ORFV002, 024 and 121 have been reported as inhibitors of the NF-κB signalling pathway, an important inflammatory signaling response to harmful cellular stimuli [14–18]. These results are consistent with our findings at early stage of infection in HFF-1 cells. Following viral replication, the host immune system was activated to stimulate the expression of the genes which invovles in innate and adaptive immunity. Among them, *CXCL10*, *CXCL11* and *CCL11* are chemokines that direct cell migration in development, immunity, inflammation and cancer [19]. NCF2, neutrophil cytosolic factor 2, is induced by IFNγ and is specifically expressed in a number of immune-cell types, including B-cells [20]. NCF2 may increase the production of the reactive oxygen species (ROX) which promote B-cell activation and differentiation to increase inflammatory cytokine response and reduce viral burden [21]. *IFNB1*, the gene of interferon-β, has antiviral, antibacterial and anticancer activities [22, 23]. DDX58 and IFNH1 are innate immune receptors that act as cytoplasmic sensors of viral nucleic acids and play a major role in sensing viral infection. They can activate a cascade of antiviral responses including the induction of type I interferons and proinflammatory cytokines. *PRKCB*, encoding protein kinase C-beta, plays a key role in B-cell activation by regulating BCR-induced NF-kappa-B activation. This data is consistent with previous reports of host gene expression after vaccinia infection [24]. In accordance with the above results, we propose that the general features of ORFV infection may be shared across cell types. Undoubtedly, the results obtained in fibroblasts may not completely reflect those observed in epithelial cells, which are the natural target cell for the virus in the host.

Interferons play a critical role as a first line of defence against viral infection. The present data shows that the type I interferon response was inhibited early in the infection, which was consistent with previous studies. Harvey et al. [25] showed that ORFV is able to block the expression of IFN stimulated genes and inhibits the JAK/STAT signaling pathway by dephosphorylating STAT1. In this study, we found differentially expressed genes, *IL12A* and *CSF3*, which are associated with the JAK/STAT signaling pathway. Orf virus inhibited the expression of *IL12A*, which can activate the members of the JAK/STAT signaling pathways and induce IFN-gamma production [26]. Another study showed that ORFV057 impaired the JAK/STAT pathway by dephosphorylating STAT1 to inhibit interferon stimulated gene expression [25]. The shorter isoform of the OV020 protein has the capability to bind double-stranded RNA (dsRNA), dsRNA-activated protein kinase (PKR), inactivate PKR, and thus act as an *in vitro* antagonist of the interferon response [27]. However, the mechanism of the suppression induced by the orf virus requires additional investigation. Interestingly, the expression of associated genes in the type I signaling pathway were up-regulated at 8 h post infection, which may contribute to the activation of host immune system. Among the DEGs, *OASL*, encoding 2’-5’-oligoadenylate synthetase-like protein, was significantly over-expressed from 3 to 8 hours post-infection (fold change=42.5, corrected p-value <0.001). OASL develops broad antiviral activity against viruses that are primarily sensed through RIG-I [28]. In this study, the associated genes (*IFIH1*, *IFNB1*, *DDX58*, *CXCL10* and *IL12A*) in the RIG-I-like receptor signaling pathway were increased obviously at 8 h post-infection(p<0.05), compared to 3 h post-infection. Viruses can manipulate the immune response of the host by various strategies and the activation of the OASL-pathway may help host cells to overcome viral escape and avoidance of innate immunity.

Infection with orf virus has critical effects in cell cycle progression (Supplementary Table 3). The expression of some genes, which are associated with cell cycle checkpoint and cell cycle arrest, were up-regulated at 8 hours post infection, including *CDK2*, *RBBP8*, *CDK1*, *KNTC1*, *CDC25A*, *BRCA1*, and *BRCA2*. It is well known that several cyclin-dependent kinases (CDKs) can be activated by CDC25A/B/C in the cell cycle. The up-regulation of CDC25A, being controlled through PI3K-Akt-mTOR signaling, can promote cell proliferation [29]. Orf virus causes localized proliferative skin lesions after infection and the lesions evolve through erythema, papule, vesicle, pustule, and scab formation [30]. The proliferative skin lesions may be attributed to the ORFV-induced promotion of the cell cycle. The ORFV poxvirus anaphase promoting complex regulator (PACR), a homologue of the subunit 11 of anaphase-promoting complex subunit (APC11), is believed to manipulate the cell cycle and enhance viral DNA synthesis by competing with APC11 for incorporation into the anaphase-promoting complex/cyclosome (APC/C) [31]. The anaphase-promoting complex plays a crucial role in cell cycle regulation, including the duration of G0/G1 and the exit from mitosis, by directing the ubiquitin-proteasome-dependent degradation of a range of proteins participating in the cell cycle [32]. The expression of PACR contributes to the creation of a stage of the cell cycle to support viral replication [12]. Meanwhile, the proliferation of infected host cells was activated when anaphase-promoting complex activity was decreased, but with an increased metabolic state. Another study shows that the VEGF-E encoded by the orf virus can regulate keratinocyte proliferation [33]. Its specific expression induces substantial intra-islet endothelial cell proliferation and the formation of hemangioma-like lesions [34]. Whether the VEGF-E protein can manipulate the cell cycle needs to be elucidated through further study. Of course, negative regulation was also activated. For instance, the human Myt1 kinase (PKMYT1) acts as a negative regulator of G2/M transition by phosphorylation of the CDK1 kinase specifically when CDK1 is complexed to cyclins and was up-regulated at 8 h.p.i. [35, 36].

Apoptotic cell death and autophagy are vital host defenses to limit viral replication and can be induced by extracellular inducers such as TNF, apoptosis stimulating fragment ligand and IFN, as well as intracellular pathways activated by macromolecular synthesis of molecules, such as viral dsRNA, after infection [12]. A range of apoptotic genes were increased with an average fold change >2 and q<0.05 after infection with orf virus. The elevated expression of the genes may have resulted in the activation of caspases that subsequently cleave a variety of cellular proteins, leading to cell death. For instance, *TNFSF10* encodes a cytokine that binds to its receptor to induce apoptosis. P53 signaling pathway was activated to regulate apoptosis by orf virus. *CCNE1*, *CCNE2*, *CDK1*, *CDK2*, *TP73*, *RRM2*, *APAF1*, *PPM1D*, and *PMAIP1* were up-regulated at 8 h.p.i. *PMAIP1* is a gene which promotes activation of caspases and apoptosis by altering the mitochondrial membrane and efflux of apoptogenic proteins. Apoptotic peptidase activating factor 1 (Apaf-1) is an important regulator of the mitochondrial apoptotic pathway to induce programmed cell death [37, 38]. In the present study, *Bcl-2*, an anti-apoptotic member of the Bcl-2 family, was up-regulated. Proteins of the Bcl-2 family play a key role in specifically regulating mitochondrial apoptosis. The up-regulation of *Bcl-2* may be associated with an anti-apoptotic factor of ORFV encoded by genes such as *ORFV125*. *ORFV125* encoded protein inhibits apoptosis by translocating to the mitochondria and blocking the release of cytochrome C [39].

In summary, this study provides an in-depth analysis of the differences in genome expression profiles of human foreskin fibroblast cells infected with orf virus strain OV-GO. A number of differential genes associated with signal transduction, cell cycle, antiviral immune response and virus-induced apoptosis were identified. A range of immune genes were up-regulated to activate the innate and adaptive immunity. Meanwhile, the cell cycle program was promoted after infection, which may be due to some ORFV immunomodulatory genes. Immunomodulatory molecules of the orf virus might affect the quality of the immune response induced by host cells. ORFV may be an effective vaccine, with some reasonable gene modifications, against a broad spectrum of pathogens and tumors.

## MATERIALS AND METHODS

### Virus and cell line

The OV-GO strain of ORFV was isolated from a goat in the Fujian province of China that had been diagnosed with orf [40, 41]. The virus strain was grown and tittered as described previously [42]. Human foreskin fibroblast (HFF-1) cell line was obtained from ATCC (Manassas, VA, USA). HFF-1 cells were cultured at 37°C in DMEM complemented with 15% FBS (Gemini, USA), containing penicillin (100 U/ml) and streptomycin (100μg/ml).

### Cell cultures, RNA extraction, mRNA-Seq

HFF-1 cells were stimulated for 0, 3 or 8 hours with live ORFV at a multiplicity of infection (MOI) of 5. Following the incubation, total RNA was extracted using TRIZOL Reagent (Life Technologies, USA), according to the manufacturer's instructions. The quantity and quality of the samples were determined on a NanoDrop ND-2000 and Agilent Bioanalyzer 2100 (RIN>9.5, 28S/18S>=1.6). Oligo-dT coated magnetic beads were used to isolate the poly-A containing mRNA molecules. Following purification, the mRNA was fragmented into small pieces and reverse transcribed into double-stranded cDNA fragments. After an end repair process and the addition of a single ‘A’ base, the cDNA fragments were ligated to the Illumina adapters. The products were purified and enriched via PCR to create the final cDNA library. The quantification and qualification of the library were carried out using Qubit® 2.0 Fluorometer and Agilent Bioanalyzer 2100 (con.=3.5-5.8ng/μL, peak length.=347-367 bp). The cDNA libraries were pooled at a concentration of 10 pM prior to clustering. Single-end read sequencing (20 million, 50 bp, single-end reads) was performed on an Illumina HiSeq2500 sequencer.

### Bioinformatics analyses of the transcriptome data

To trim adapters, PCR primer sequences, ribosome RNA and low quality base reads, the raw RNA-seq reads were processed with the FASTX-Toolkit. Reads with a length less than 25 nt were discarded. The clean reads were mapped to the hg19 human reference genome (http://hgdownload.cse.ucsc.edu/goldenPath/hg19/bigZips/chromFa.tar.gz) using TopHat version 2, allowing for 2 nt of mismatch [43]. For a better comparison of the gene expression levels across different genes and different samples, the counts of each unigene in each sample were normalized using trimmed mean of M-values (TMM). FPKM (fragments per kilobase of exon model per million mapped reads) value was used to calculate the expression level for each gene [44–46]. The data were normalized to the RNA-seq data from the HFF-1 samples without incubation with orf virus (or samples 3 hours post-infection.). False discovery rates were computed by the EdgeR package, which was used to estimate the common dispersion [47]. Genes with a q-value (corrected p-value) less than 0.05 and a fold change greater than 2 were categorized as differentially expressed. All the identified differentially expressed genes (DEGs) were mapped to the terms in GO database to analyze functional significance. The DEGs were also mapped to terms in the KEGG PATHWAY Database for pathway analysis. The principle component analysis (PCA), cluster and trend analyses were performed using the OmicShare tools, a free online platform for data analysis (www.omicshare.com/tools).

### Quantitative real-time RT-PCR

The total RNA for each sample was used to prepare cDNA with the cDNA Synthesis Kit (Biotool, USA), according to the manufacturer's protocol. Real-time quantitative PCR (RT-PCR) was performed using the TransStart Tip Green qPCR SuperMix Kit (Transgen, China), according to the manufacturer's instructions, with the appropriate primers (Supplementary Table 5). Briefly, the reaction protocol was 30 s at 94°C, 43 cycles of 5 s at 94 °C and 34 s at 60 °C. The relative quantitation was performed using the ΔΔCt method and the expression of detectable genes was normalized to the reference gene β-actin. The qPCR experiments were carried out in quadruplicate with 3 independent repeats. Expression level of the genes are shown as the normalized cycle threshold (ΔCt = Ct target gene – Ct reference gene, ΔΔCt = ΔCt post-infection – ΔCt un-infected) [48].

### Western blots

Western blots were used to assess the expression level of ORFV protein. HFF-1 cells were incubated with ORFV at 0, 6, 12, 24, 36, and 48 h. The infected cells were lysed in 1× loading buffer and boiled for 15min before centrifugation. The samples were resolved by SDS-PAGE in 10% gels, followed by blotting to PVDF membranes. Then the membranes were blocked with 5% skim milk and probed with antibody anti-ORFV086 and anti-ORFV079. After washing, the blots were incubated with secondary goat anti-rabbit IgG-(HRP). Finally, they were washed and developed using a chemiluminescent substrate.

### Statistical analysis

An empirical Bayesian analysis was used to shrink the dispersions towards a consensus value, effectively borrowing information between genes [46, 49]. Differential expression was assessed for each gene using an exact test analogous to Fisher's exact test [46, 50]. Genes with a q-value lower than 0.05 and with a fold change greater than 2 were considered differentially expressed.

## SUPPLEMENTARY MATERIALS FIGURES AND TABLES










