# Supplementary material for: Transcriptomic profiles of human foreskin fibroblast cells in response to orf virus
Source: Oncotarget. 2017 Apr 25;8(35):58668–85. doi: 10.18632/oncotarget.17417 (PMC5601683; doi:10.18632/oncotarget.17417)
Supplement: Supplementary file 1 [file oncotarget-08-58668-s001.pdf]

Transcriptomic profiles of human foreskin fibroblast cells in response to orf virus

SUPPLEMENTARY FIGURES AND TABLES

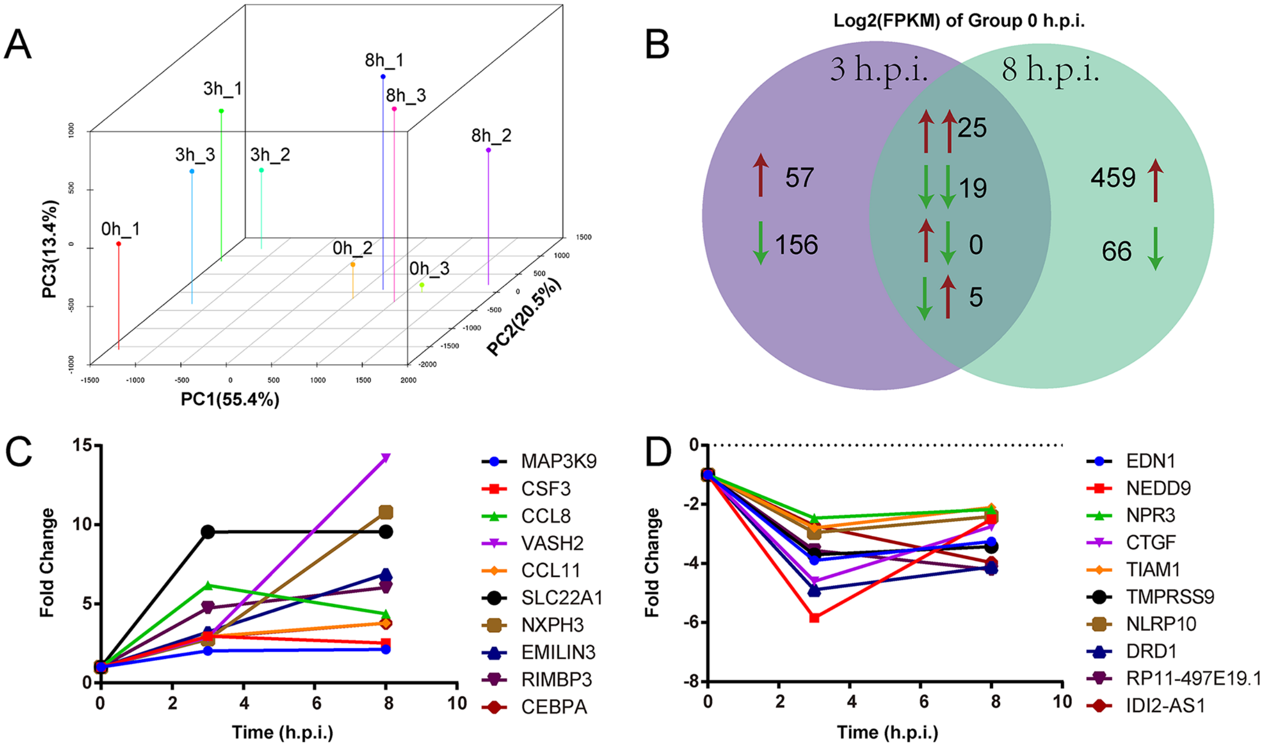

Supplementary Figure 1: Principal components analysis (PCA) of the transcriptome data and data analyses between the 3 h and 8 h post-infection groups. (A) PCA of the ORFV transcriptome data. Infection period appeared to be the major source of variation, as indicated by red ellipsoids. **(B)** The number of differentially expressed genes at 3 and 8 h post-infection is shown in the Venn diagram, compared to the uninfected group. The upward red arrows represent the up-regulated genes and downward green arrows represent the down-regulated genes. Pairs of arrows in the intersection refer to the direction of fold change on the left (3 h.p.i.) and right (8 h.p.i.) sides. Representative expression patterns of genes that were up- **(C)** or down- **(D)** regulated over time.

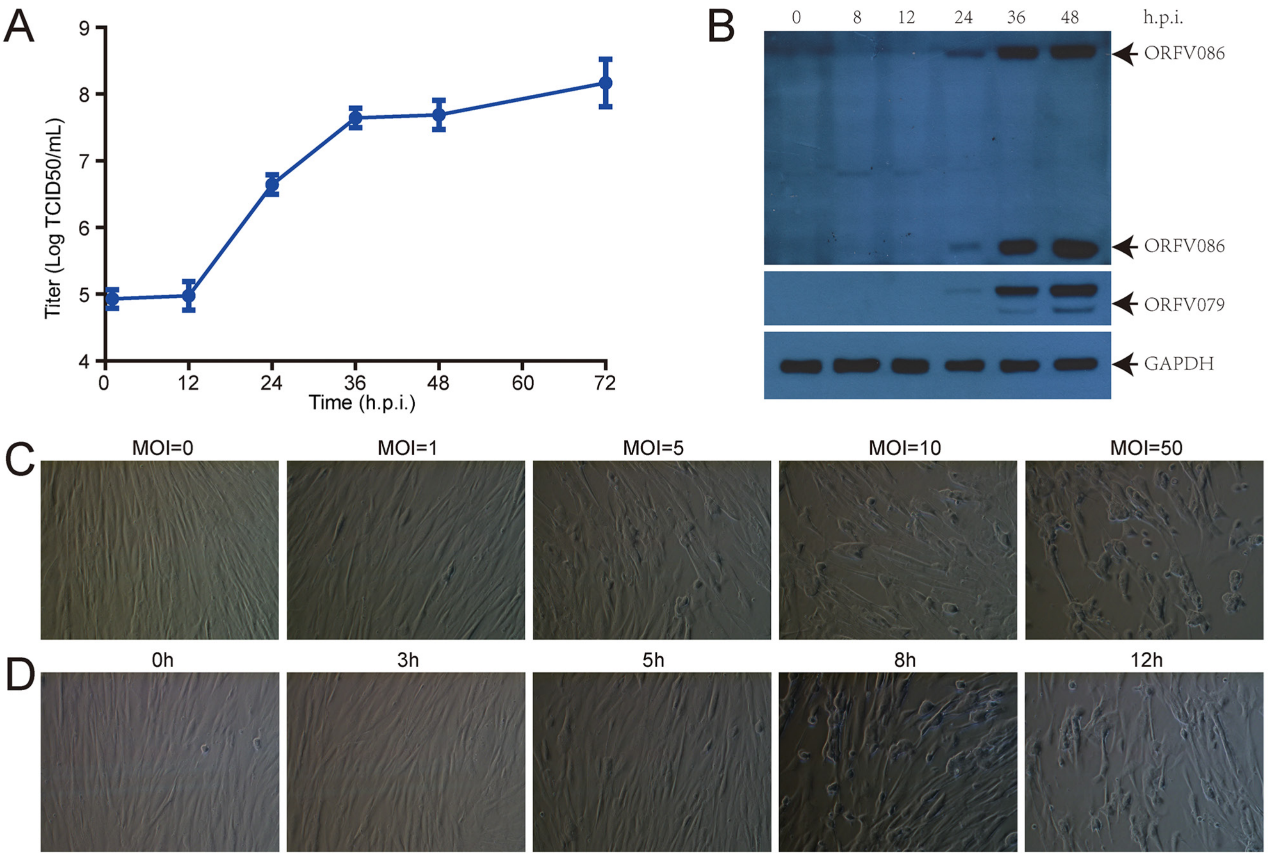

Supplementary Figure 2: Replication characteristics and CPE of OV-GO in HFF-1 cells. **(A)** One step growth curves were performed in HFF-1 cells with OV-GO (MOI=5) over the indicated time periods. Data represent the averages of the results of three independent experiments. Error bars show standard deviations. **(B)** The accumulation of ORFV079 and ORFV086 in HFF-1 cells is increased at 24 h.p.i. HFF-1 cells were infected with OV-GO (MOI=5) over the indicated time periods, and the whole-cell lysates were subjected to SDS-PAGE and analyzed by immunoblotting with the indicated antibodies. **(C)** Morphological changes in HFF-1 cells infected with ORFV at MOI of 0, 1, 5, 10 and 50 in six-well plates; changes were examined by phase-contrast microscopy at 8 hours post infection. **(D)** HFF-1 cells were incubated with ORFV (5 PFU/cell) and examined at 0, 3, 5, 8 and 12 hours post infection.

Supplementary Table 1: GO analysis of DEGs unique to type I interferon response between two groups

GO_term	Number of up_genes	Number of down_genes	P value
3 h.p.i.vs 0 h.p.i.			
type I interferon signaling pathway	0	5	1.33E-04
response to type I interferon	0	5	1.33E-04
8 h.p.i.vs 0 h.p.i.			
negative regulation of type I interferon production	3	1	4.69E-03
regulation of type I interferon production	5	1	1.73E-02
positive regulation of type I interferon production	4	0	4.69E-02
type I interferon production	5	1	1.73E-02
8 h.p.i.vs 3 h.p.i.			
type I interferon production	7	0	4.95E-02
response to type I interferon	7	0	7.24E-03
positive regulation of type I interferon production	6	0	2.58E-02
regulation of type I interferon production	7	0	4.95E-02
type I interferon signaling pathway	7	0	7.24E-03

Supplementary Table 2: Functions of DEGs associated with growth and death.

See Supplementary File 1

Supplementary Table 3: Expression levels of genes associated with cell cycle.

See Supplementary File 2

Supplementary Table 4: Functions of DEGs associated with immune response.

See Supplementary File 3

Supplementary Table 5: Primers used for quantitative real-time PCR.

See Supplementary File 4
