# Supplementary material for: Transcriptomic profiles of human foreskin fibroblast cells in response to orf virus
Source: Oncotarget. 2017 Apr 25;8(35):58668–85. doi: 10.18632/oncotarget.17417 (PMC5601683; doi:10.18632/oncotarget.17417)
Supplement: Supplementary file 2 [file oncotarget-08-58668-s002.docx]

| **Supplementary Table 2: Functions of DEGs accociated with growth and death** | | | | |
| --- | --- | --- | --- | --- |
| Genes | Fold change (p-value) | | | The functions of DEGs from STRING10,wikipedia or interference |
|  | 3 h.p.i. vs. 0 h.p.i. | 8 h.p.i. vs. 0 h.p.i. | 8 h.p.i. vs. 3 h.p.i. |  |
| CDC25B | 2.01(2.87E-48) | 1.20(3.37E-01) | -1.69 （4.30E-15） | Acts as a central target and regulator of the G2/M checkpoint mechanisms when activated in response to DNA injury. |
| TP73 | -1.30(9.95E-01) | 5.54(1.41E-02) | 7.23 （1.23E-03） | Acts as a tumor suppressor in many tumor types and induces growth arrest or apoptosis. |
| APAF1 | 1.6(3.72E-08) | 2.30(1.01E-13) | 1.43 （2.66E-03） | Acts as an important regulator of the mitochondrial apoptotic pathway to induce programmed cell death. |
| PKMYT1 | 1.50(1.00E-03) | 2.40(1.51E-12) | 1.59 （1.29E-03） | Acts as a negative regulator of G2/M transition by phosphorylation of the CDK1 kinase specifically when CDK1 is complexed to cyclins. |
| MCM2 | 1.40(1.42E-07) | 2.74(1.78E-21) | 1.96 （9.42E-14） | Acts as component of the MCM2-7 complex which is the putative replicative helicase essential for once per cell cycle' DNA replication initiation and elongation in eukaryotic cells. |
| MCM3 | 1.13(1.00E-03) | 2.95(2.26E-30) | 2.40 （1.56E-29） |  |
| MCM4 | 1.12(1.49E-01) | 2.84(7.79E-30) | 2.54 （2.73E-30） |  |
| MCM5 | 1.33(1.94E-05) | 2.47(1.73E-19) | 1.85 （5.20E-11） |  |
| MCM6 | 1.03(6.75E-01) | 2.27(4.26E-17) | 2.20 （1.30E-22） |  |
| MCM7 | 1.30(3.51E-05) | 2.11(1.27E-13) | 1.63 （4.09E-07） |  |
| E2F1 | 1.61(2.74E-09) | 3.55(1.93E-28) | 2.20 （2.83E-15） | The DRTF1/E2F complex functions in the control of cell-cycle progression from G1 to S phase. It can mediate both cell proliferation and TP53/p53-dependent apoptosis. |
| CDC45 | 1.34(3.00E-03) | 2.90(7.53E-19) | 2.17 （3.21E-13） | Required for initiation of chromosomal DNA replication [1]. |
| PCNA | 1.06(2.95E-01) | 2.64(5.35E-25) | 2.48 （8.31E-33） | It is involved in the control of eukaryotic DNA replication and plays a key role in DNA damage response. |
| CDC7 | -1.06(9.33E-01) | 2.72(1.09E-13) | 2.89 （8.56E-18） | It seems to phosphorylate critical substrates that regulate the G1/S phase transition and/or DNA replication. |
| CCNE2 | -1.05(9.97E-01) | 7.61(4.36E-36) | 7.98 （4.89E-70） | Essential for the control of the cell cycle at the late G1 and early S phase. |
| PARP2 | 1.14(1.86E-01) | 2.31(8.58E-14) | 2.03 （2.01E-12） | Involved in the base excision repair (BER) pathway, by catalyzing the poly(ADP-ribosyl)ation of a limited number of acceptor proteins involved in chromatin architecture and in DNA metabolism. |
| PPM1D | 1.19(4.60E-02) | 2.13(2.68E-11) | 1.78 （8.53E-09） | Required for the relief of p53-dependent checkpoint mediated cell cycle arrest. |
| RRM2 | 1.03(7.20E-01) | 2.27(3.94E-18) | 2.20 （1.63E-20） | Provides the precursors necessary for DNA synthesis. |
| ORC1 | 1.34(1.30E-02) | 5.26(4.29E-41) | 3.92 （1.52E-42） | Component of the origin recognition complex (ORC) that binds origins of replication.ORC is required to assemble the pre-replication complex necessary to initiate DNA replication. |
| CDC25A | 1.36(8.24E-03) | 4.7(5.67E-43) | 3.45 （4.95E-33） | CDC25A is required for progression from G1 to the S phase of the cell cycle, but also plays roles in later cell cycle events.CDC25A is specifically degraded in response to DNA damage, resulting in cell cycle arrest. |
| CDK2 | 1.21(7.40E-03) | 2.54(9.96E-21) | 2.11 （1.22E-18） | Acts at the G1-S transition to promote the E2F transcriptional program and the initiation of DNA synthesis, and modulates G2 progression; controls the timing of entry into mitosis/meiosis by controlling the subsequent activation of cyclin B/CDK1 by phosphorylation. |
| CDC6 | 1.32(1.49E-04) | 5.48(4.65E-65) | 4.16 （4.04E-76） | Involved in the initiation of DNA replication. Also participates in checkpoint controls that ensure DNA replication is completed before mitosis is initiated. |
| BCL2 | -1.05(1) | 3.70(1.76E-15) | 3.87 （3.52E-18） | Suppresses apoptosis in a variety of cell systems. Regulates cell death by controlling the mitochondrial membrane permeability. |
| LMNB1 | -1.10(4.33E-01) | 2.55(5.87E-22) | 2.82 （3.10E-36） | Lamin proteins are thought to be involved in nuclear stability, chromatin structure, and gene expression. |
| SKP2 | -1.11(3.34E-01) | 1.80(4.34E-08) | 2.00 （5.19E-15） | Involved in the ubiquitination and subsequent proteasomal degradation of target proteins. |
| SPDYA | 1.82(1) | 8.09(5.61E-03) | 4.45 （7.18E-03） | Regulates the G1/S phase transition of the cell cycle by binding and activating CDK1, CDK2 and CDKN1B/KIP1. |
| CCNE1 | 1.39(7.77E-04) | 3.25(1.95E-21） | 2.34 （3.45E-16） | Essential for the control of the cell cycle at the G1/S (start) transition. |
| BCL2L11 | 1.55 （1.19E-02） | 3.51(2.73E-17) | 2.27 （7.19E-10） | Apoptosis facilitator [2]. |
| FOS | -4.35(1.42E-13) | -1.05(9.90E-01) | 4.14 （4.89E-10） | Has a critical function in regulating the development of cells destined to form and maintain the skeleton. |
| SMC3 | -1.54(2.88E-07) | 1.45(1.33E-01) | 2.22 （2.51E-07） | It is the central component of cohesin, a complex required for chromosome cohesion during the cell cycle. |
| TTK | -1.45(5.53E-04) | 1.43(9.6E-02) | 2.07 （5.70E-07） | Probably associated with cell proliferation. |
| SPDYE2 | -1.67(6.40E-02) | 1.53(1.43E-01) | 2.55 （8.36E-06） | No items. |
| SGOL1 | -1.08(9.87E-01) | 2.37(1.58E-04) | 2.56 （8.00E-07） | Plays a central role in chromosome cohesion during mitosis. |
| TNFSF10 | -1.79(6.35E-01) | 3.64(0.00125334) | 6.55 （2.57E-07） | Induces apoptosis. |
| CDK1 | -1.33(3.51E-03) | 2.07(2.84E-07) | 2.74 （8.55E-19） | Plays a key role in the control of the eukaryotic cell cycle by modulating the centrosome cycle as well as mitotic onset. It promotes G2-M transition, and regulates G1 progress and G1-S transition via association with multiple interphase cyclins. |
| RBL1 | -1.39(7.20E-03) | 2.38(1.04E-10) | 3.29 （4.21E-23） | Key regulator of entry into cell division. |
| FBXO5 | -1.39(4.00E-03) | 2.17(2.32E-09) | 3.02 （2.13E-21） | Regulates progression through early mitosis by inhibiting the anaphase promoting complex/cyclosome (APC). |
| PMAIP1 | -1.47(5.94E-06) | 1.73(6.06E-06) | 2.53 （2.90E-20） | Promotes activation of caspases and apoptosis. |
| References | | | | |
| 1 Kohler C, Koalick D, Fabricius A, Parplys AC, Borgmann K, Pospiech H and Grosse F. Cdc45 is limiting for replication initiation in humans. CELL CYCLE. 2016; 15(7):974-985. | | | | |
| 2 Yang D, Okamura H, Teramachi J and Haneji T. Histone demethylase Jmjd3 regulates osteoblast apoptosis through targeting anti-apoptotic protein Bcl-2 and pro-apoptotic protein Bim. Biochim Biophys Acta. 2016; 1863(4):650-659. | | | | |
