# Supplementary material for: Transcriptomic profiles of human foreskin fibroblast cells in response to orf virus
Source: Oncotarget. 2017 Apr 25;8(35):58668–85. doi: 10.18632/oncotarget.17417 (PMC5601683; doi:10.18632/oncotarget.17417)
Supplement: Supplementary file 3 [file oncotarget-08-58668-s003.docx]

| **Supplemental Table 3. Expression levels of genes associated with cell cycle** | | | | | | | | |
| --- | --- | --- | --- | --- | --- | --- | --- | --- |
| gene name | 3 h.p.i. vs. 0 h.p.i. | |  | 8 h.p.i. vs. 0 h.p.i. | |  | 8 h.p.i. vs. 3 h.p.i. | |
|  | FC | q-value |  | FC | q-value |  | FC | q-value |
| G1 Phase and G1/S Transition | | | | | | | | |
| E2F1 | 1.61 | 2.74E-09 |  | 3.55 | 1.93E-28 |  | 2.20 | 2.83E-15 |
| SKP2 | -1.11 | 3.34E-01 |  | 1.80 | 4.34E-08 |  | 2.00 | 5.19E-15 |
| CCNE1 | 1.39 | 7.77E-04 |  | 3.25 | 1.95E-21 |  | 2.34 | 3.45E-16 |
| CDC25A | 1.36 | 8.24E-03 |  | 4.70 | 5.67E-43 |  | 3.45 | 4.95E-33 |
| S Phase and DNA Replication | | | | | | | | |
| MCM4 | 1.12 | 1.49E-01 |  | 2.84 | 7.79E-30 |  | 2.54 | 2.73E-30 |
| MCM3 | 1.23 | 1.34E-03 |  | 2.95 | 2.26E-30 |  | 2.40 | 1.56E-29 |
| MCM5 | 1.33 | 1.94E-05 |  | 2.47 | 1.73E-19 |  | 1.85 | 5.20E-11 |
| MCM2 | 1.40 | 1.42E-07 |  | 2.74 | 1.78E-21 |  | 1.96 | 9.42E-14 |
| CDC6 | 1.32 | 1.49E-04 |  | 5.48 | 4.65E-65 |  | 4.16 | 4.04E-76 |
| CDC25B | 2.01 | 2.87E-48 |  | 1.19 | 3.37E-01 |  | -1.68 | 4.30E-15 |
| G2 Phase and G2/M Transition | | | | | | | | |
| CDC25A | 1.36 | 8.24E-03 |  | 4.70 | 5.67E-43 |  | 3.45 | 4.95E-33 |
| CDC25B | 2.01 | 2.87E-48 |  | 1.19 | 3.37E-01 |  | -1.68 | 4.30E-15 |
| CDK5R1 | 1.01 | 1.00E+00 |  | 3.68 | 3.89E-10 |  | 3.64 | 3.97E-15 |
| M Phase |  |  |  |  |  |  |  |  |
| CDC6 | 1.32 | 1.49E-04 |  | 5.48 | 4.65E-65 |  | 4.16 | 4.04E-76 |
| RAD51 | 1.35 | 5.85E-02 |  | 3.31 | 3.01E-22 |  | 2.46 | 1.60E-11 |
| MRE11A | -1.36 | 3.26E-02 |  | 1.52 | 8.13E-02 |  | 2.07 | 3.38E-05 |
| CDK1 | -1.33 | 3.51E-03 |  | 2.07 | 2.84E-07 |  | 2.74 | 8.55E-19 |
| Cell Cycle Checkpoint and Cell Cycle Arrest | | | | | | | | |
| CDK2 | 1.21 | 7.44E-03 |  | 2.54 | 9.96E-21 |  | 2.11 | 1.22E-18 |
| RBBP8 | -1.30 | 3.00E-03 |  | 2.15 | 2.48E-09 |  | 2.79 | 4.25E-22 |
| CDK1 | -1.33 | 3.51E-03 |  | 2.07 | 2.84E-07 |  | 2.74 | 8.55E-19 |
| KNTC1 | -1.48 | 8.81E-06 |  | 1.75 | 7.52E-05 |  | 2.59 | 9.84E-17 |
| CDC25A | 1.36 | 8.24E-03 |  | 4.70 | 5.67E-43 |  | 3.45 | 4.95E-33 |
| BRCA1 | -1.45 | 5.93E-04 |  | 3.16 | 1.38E-15 |  | 4.60 | 5.31E-34 |
| BRCA2 | -1.93 | 1.53E-03 |  | 2.90 | 1.36E-05 |  | 5.59 | 1.03E-13 |
| Regulation of Cell Cycle | | | | | | | | |
| CDK2 | 1.21 | 7.44E-03 |  | 2.54 | 9.96E-21 |  | 2.11 | 1.22E-18 |
| CDC6 | 1.32 | 1.49E-04 |  | 5.48 | 4.65E-65 |  | 4.16 | 4.04E-76 |
| E2F1 | 1.61 | 2.74E-09 |  | 3.55 | 1.93E-28 |  | 2.20 | 2.83E-15 |
| SKP2 | -1.11 | 3.34E-01 |  | 1.80 | 4.34E-08 |  | 2.00 | 5.19E-15 |
| CDK1 | -1.33 | 3.51E-03 |  | 2.07 | 2.84E-07 |  | 2.74 | 8.55E-19 |
| CCNE1 | 1.39 | 7.77E-04 |  | 3.25 | 1.95E-21 |  | 2.34 | 3.45E-16 |
| KNTC1 | -1.48 | 8.81E-06 |  | 1.75 | 7.52E-05 |  | 2.59 | 9.84E-17 |
| BRCA2 | -1.93 | 1.53E-03 |  | 2.90 | 1.36E-05 |  | 5.59 | 1.03E-13 |
| BCL2 | -1.05 | 1.00E+00 |  | 3.70 | 1.76E-15 |  | 3.87 | 3.52E-18 |
| CDK5R1 | 1.01 | 1.00E+00 |  | 3.68 | 3.89E-10 |  | 3.64 | 3.97E-15 |
| Negative Regulation of Cell Cycle | | | | | | | | |
| RBL1 | -1.38 | 7.22E-03 |  | 2.38 | 1.04E-10 |  | 3.29 | 4.21E-23 |
| BRCA1 | -1.45 | 5.93E-04 |  | 3.16 | 1.38E-15 |  | 4.60 | 5.31E-34 |
