# Supplementary material for: Transcriptomic profiles of human foreskin fibroblast cells in response to orf virus
Source: Oncotarget. 2017 Apr 25;8(35):58668–85. doi: 10.18632/oncotarget.17417 (PMC5601683; doi:10.18632/oncotarget.17417)
Supplement: Supplementary file 4 [file oncotarget-08-58668-s004.docx]

| **Supplementary Table 4: Functions of DEGs accociated with immune response** | | | | |
| --- | --- | --- | --- | --- |
| Genes name | Fold change (p-value) | | | Immune function from STRING10 (http://string-db.org/) or interference |
|  | 3 h.p.i. vs. 0 h.p.i. | 8 h.p.i. vs. 0 h.p.i. | 8 h.p.i. vs. 3 h.p.i. |  |
| TNFSF13B | -2.44 (2.43E-02) | -1.15 (8.92E-01) | 2.13 (6.53E-02) | Cytokine that involved in the stimulation of B- and T-cell function and the regulation of humoral immunity. |
| FOS | -4.35 (1.42E-13) | -1.05 (9.90E-01) | 4.14 (4.89E-10) | The transcription factors that involved in recruitment and activation of immune cells [1]. |
| POLR3G | -2.17 (2.70E-03) | -1.01 (1) | 2.12 (2.96E-03) | It catalyzes the transcription of DNA into RNA. |
| PRKCE | -2.17 (4.67E-25) | -1.06 (7.34E-01) | 2.05 (1.73E-12) | Play a key role in macrophage and dendritic cell (DC) activation in response to LPS [2]. |
| MAP3K8 | -2.44 (1.93E-14) | -1.45 (2.86E-02) | 1.68 (3.67E-03) | Required for TLR4 activation of the MEK/ERK pathway and able to activate NF-kappa-B 1 by stimulating proteasome-mediated proteolysis of NF-kappa-B 1/p105. |
| TLR3 | -2.33 (4.49E-06) | -1.18 (7.18E-01) | 1.99 (4.73E-03) | Key component of innate and adaptive immunity.Acts via the adapter TRIF/TICAM1, leading to NF-kappa-B activation, cytokine secretion and the inflammatory response |
| KIT | -2.00(8.92E-14) | -1.04 (8.82E-01) | 1.93 (2.17E-07) | Act as cell-surface receptor for the cytokine KITLG/SCF and play an essential role in the regulation of cell survival and proliferation. |
| IL12A | -2.94 (6.98E-04) | -1.30 (0.6.15E-01) | 2.26 (3.35E-02) | Cytokine that can act as a growth factor for activated T and NK cells, enhance the lytic activity of NK/lymphokine- activated Killer cells, and stimulate the production of IFN-gamma by resting PBMC |
| TNFAIP3 | -1.35 (1.14E-05) | -2.27 (1.98E-11) | -1.67 (1.49E-07) | Involved in immune and inflammatory responses signaled by cytokines or pathogens via Toll-like receptors (TLRs) through terminating NF-kappa-B activity. |
| ULBP1 | -2.86 (1.09E-02) | -2.38 (4.53E-02) | 1.20 (1) | Ligand for the NKG2D receptor and to activate multiple signaling pathways in primary NK cells, resulting in the production of cytokines and chemokines. |
| TIAM1 | -2.78 (3.49E-03) | -2.08 (4.71E-02) | 1.33 (7.63E-01) | Modulates the activity of RHO-like proteins and connects extracellular signals to cytoskeletal activities. |
| ITGB7 | 2.86 (1.89E-02) | 1.62 (7.28E-01) | -1.75 (1.03E-01) | An adhesion molecule that mediates lymphocyte migration and homing to gut-associated lymphoid tissue (GALT). |
| THBD | 2.87 (1.30E-02) | 1.02(1) | -2.78(3.63E-03) | Thrombomodulin links coagulation to inflammation and immunity [3]. |
| IFNB1 | 0 | Inf(2.29E-03) | Inf(8.65E-05) | Produced by monocytes and macrophages and is known for antiviral,antibacterial and anticancer activities |
| ARRB2 | 1.34(3.09E-03) | 2.25 (7.17E-11) | 1.68 (8.27E-06) | It can inhibit proinflammatory chemokine production and attenuates inflammation [4]. |
| MB21D1 | 1.30 (5.45E-02) | 3.53 (3.08E-22) | 2.71 (1.25E-19) | Has antiviral activity by acting as a key cytosolic DNA sensor, the presence of DNA in the cytoplasm being a danger signal that triggers the immune responses. |
| AMPH | -1.06 (9.37E-01) | 2.06 (6.48E-06) | 2.22 (1.61E-06) | Participates in phagocytosis and is transiently associated with early phagosomes [5, 6]. |
| CXCL10 | -1.85 (7.12E-02) | 3.81 (3.63E-08) | 7.12 (1.15E-17) | It is secreted by leukocytes and tissue cells and chemotactic for monocytes and T-lymphocytes [7, 8]. |
| TNFSF10 | -1.79 (6.35E-01) | 3.64 (1.25E-03) | 6.55 (2.57E-07) | Induces apoptosis. |
| DDX58 | -1.49(6.49E-04) | 2.05 (2.29E-05) | 3.06 (3.90E-22) | Innate immune receptor which acts as a cytoplasmic sensor of viral nucleic acids and plays a major role in sensing viral infection and in the activation of a cascade of antiviral responses including the induction of type I interferons and proinflammatory cytokines. |
| IFIH1 | -1.59 (2.97E-03) | 2.28 (1.39E-05) | 3.64 (1.78E-18) | Innate immune receptor which acts as a cytoplasmic sensor of viral nucleic acids and plays a major role in sensing viral infection and in the activation of a cascade of antiviral responses including the induction of type I interferons and proinflammatory cytokines. |
| GUCY1B3 | -1.27 (5.28E-01) | 2.46 (3.07E-06) | 3.10 (6.09E-09) | It is associated with systemic inflammatory response [9]. |
| CXCL11 | 2.15 (9.03E-01) | 14.81 (8.23E-06) | 6.88 (2.27E-05) | Chemotactic for interleukin-activated T-cells but not unstimulated T-cells, neutrophils or monocytes. |
| NCF2 | 3.13 (5.28E-02) | 3.98 (3.16E-02) | 1.27 (8.20E-01) | It involves in the pathogenesis of inflammatory-related common diseases [10]. |
| PRKCB | 2.91 (2.14E-01) | 5.24 (1.20E-02) | 1.80 (2.61E-01) | Plays a key role in B-cell activation by regulating BCR-induced NF-kappa-B activation. |
| CCL11 | 2.93 (6.52E-11) | 3.78 (2.21E-10) | 1.29 (3.20E-01) | In response to the presence of allergens, this protein directly promotes the accumulation of eosinophils, a prominent feature of allergic inflammatory reactions. |
| CSF3 | 2.95 (8.43E-15) | 2.51 (1.10E-06) | -1.18(2.84E-01) | Granulocyte/macrophage colony-stimulating factors are cytokines that act in hematopoiesis by controlling the production, differentiation, and function of 2 related white cell populations of the blood, the granulocytes and the monocytes-macrophages. |
| CCL8 | 6.17 (5.91E-05) | 4.37 (2.47E-02) | -1.14 (4.01E-01) | Chemotactic factor that attracts monocytes, lymphocytes, basophils and eosinophils. May play a role in inflammatory host responses. |
| References | | | | |
| 1 Uluckan O, Guinea-Viniegra J, Jimenez M and Wagner EF. Signalling in inflammatory skin disease by AP-1 (Fos/Jun). CLIN EXP RHEUMATOL. 2015; 33(4 Suppl 92):S44-S49. | | | | |
| 2 Aksoy E, Goldman M and Willems F. Protein kinase C epsilon: a new target to control inflammation and immune-mediated disorders. Int J Biochem Cell Biol. 2004; 36(2):183-188. | | | | |
| 3 Morser J. Thrombomodulin links coagulation to inflammation and immunity. CURR DRUG TARGETS. 2012; 13(3):421-431. | | | | |
| 4 Gaffal E, Jakobs M, Glodde N, Schroder R, Kostenis E and Tuting T. beta-arrestin 2 inhibits proinflammatory chemokine production and attenuates contact allergic inflammation in the skin. J INVEST DERMATOL. 2014; 134(8):2131-2137. | | | | |
| 5 Gold ES, Morrissette NS, Underhill DM, Guo J, Bassetti M and Aderem A. Amphiphysin IIm, a novel amphiphysin II isoform, is required for macrophage phagocytosis. IMMUNITY. 2000; 12(3):285-292. | | | | |
| 6 Gold ES, Simmons RM, Petersen TW, Campbell LA, Kuo CC and Aderem A. Amphiphysin IIm is required for survival of Chlamydia pneumoniae in macrophages. J EXP MED. 2004; 200(5):581-586. | | | | |
| 7 Romagnani P and Crescioli C. CXCL10: a candidate biomarker in transplantation. CLIN CHIM ACTA. 2012; 413(17-18):1364-1373. | | | | |
| 8 van den Borne P, Quax PH, Hoefer IE and Pasterkamp G. The multifaceted functions of CXCL10 in cardiovascular disease. BIOMED RES INT. 2014; 2014:893106. | | | | |
| 9 Geiger EV, Maier M, Schiessling S, Wutzler S, Lehnert M, Marzi I and Henrich D. Subsequent gene expression pattern in dendritic cells following multiple trauma. Langenbecks Arch Surg. 2013; 398(2):327-333. | | | | |
| 10 Cunninghame GD, Morris DL, Bhangale TR, Criswell LA, Syvanen AC, Ronnblom L, Behrens TW, Graham RR and Vyse TJ. Association of NCF2, IKZF1, IRF8, IFIH1, and TYK2 with systemic lupus erythematosus. PLOS GENET. 2011; 7(10):e1002341. | | | | |
