# Supplementary material for: Transcriptomic profiles of human foreskin fibroblast cells in response to orf virus
Source: Oncotarget. 2017 Apr 25;8(35):58668–85. doi: 10.18632/oncotarget.17417 (PMC5601683; doi:10.18632/oncotarget.17417)
Supplement: Supplementary file 5 [file oncotarget-08-58668-s005.docx]

| Supplemental Table 5. Primers used for quantitative real-time PCR | | | | |
| --- | --- | --- | --- | --- |
| mRNA | Forward primer | Reverse primer | length | Reference |
| CCL8 | TGGAGAGCTACACAAGAATCACC | TGGTCCAGATGCTTCATGGAA | 133bp | Primer bank ID:22538815c1 |
| TNFRSF1B | CGGGCCAACATGCAAAAGTC | CAGATGCGGTTCTGTTCCC | 178bp | Primer bank ID:23312365c1 |
| CCL11 | CCCTTCAGCGACTAGAGAGC | CAGCTTTCTGGGGACATTTG | 60bp | [1] |
| PLCB2 | ATCCGGGATACTCGCTTTGG | CACCACCGTGAGTGTCTTCAG | 117bp | Primer bank ID:95147332c1 |
| BCL-2 | GGTGGGGTCATGTGTGTGG | CGGTTCAGGTACTCAGTCATCC | 89bp | Primer bank ID:72198345c1 |
| SOCS3 | CCCTCGCCACCTACTGAA | TCCGACAGAGATGCTGAAGA | 175bp | [2] |
| EDN1 | ACTTCTGCCACCTGGACATCA | CTCCAAGGCTCTCTTGGACCTA | 92bp | [3] |
| TNFAIP3 | CTGGGACCATGGCACAACTC | CGGAAGGTTCCATGGGATTC | 181bp | [4] |
| SSTR1 | CCAGCATCTACTGTCTGACTGT | ATGACGAGCAGCGATAGCAC | 139bp | Primer bank ID:33946330c2 |
| TIAM1 | AAGACGTACTCAGGCCATGTCC | GACCCAAATGTCGCAGTCAG | 253bp | [5] |
| NEDD9 | CGGCAGTTGCTGTGCTTCTA | AATGGCGTTGAGAAGGGAAA | 66bp | [[6]](http://www.ncbi.nlm.nih.gov/pubmed/24699941) |
| CTGF | GGAAAAGATTCCCACCCAAT | TGCTCCTAAAGCCACACCTT | 153bp | [7] |
| IL12A | TGGCCCTGTGCCTTAGTAGT | CAGAAGCTTTGCATTCATGG | 80bp | [8] |
| FOS | ATGGGCTCGCCTGTCAAC | CAGTGACCGTGGGAATGAAGTT | 79bp | [9] |
| CSF3 | GCTGCTTGAGCCAACTCCATA | GAACGCGGTACGACACCTC | 285bp | Primer bank ID:296011056c1 |
| Beta-actin | GATCATTGCTCCTCCTGAGC | ACTCCTGCTTGCTGATCCAC | 101bp | [10] |
| References | | | | |
| 1 Diel DG, Luo S, Delhon G, Peng Y, Flores EF and Rock DL. A nuclear inhibitor of NF-kappaB encoded by a poxvirus. J VIROL. 2011; 85(1):264-275. | | | | |
| 2 Attia FM, Hassan AM, El-Maraghy NN and Ibrahium GH. Clinical significance of suppressor of cytokines signalling-3 mRNA expression from patients with non-Hodgkin lymphoma under chemotherapy. CANCER BIOMARK. 2011; 11(1):41-47. | | | | |
| 3 Grosse J, Wehland M, Pietsch J, Ma X, Ulbrich C, Schulz H, Saar K, Hubner N, Hauslage J, Hemmersbach R, Braun M, van Loon J, Vagt N, Infanger M, Eilles C and Egli M, et al. Short-term weightlessness produced by parabolic flight maneuvers altered gene expression patterns in human endothelial cells. FASEB J. 2012; 26(2):639-655. | | | | |
| 4 Zhu L, Zhang F, Shen Q, Chen S, Wang X, Wang L, Yang L, Wu X, Huang S, Schmidt CA and Li Y. Characteristics of A20 gene polymorphisms in T-cell acute lymphocytic leukemia. HEMATOLOGY. 2014; 19(8):448-454. | | | | |
| 5 Yeh CS, Wang JY, Wu CH, Chong IW, Chung FY, Wang YH, Yu YP and Lin SR. Molecular detection of circulating cancer cells in the peripheral blood of patients with colorectal cancer by using membrane array with a multiple mRNA marker panel. INT J ONCOL. 2006; 28(2):411-420. | | | | |
| 6 Chou CP, Huang NC, Jhuang SJ, Pan HB, Peng NJ, Cheng JT, Chen CF, Chen JJ and Chang TH. Ubiquitin-conjugating enzyme UBE2C is highly expressed in breast microcalcification lesions. PLOS ONE. 2014; 9(4):e93934. | | | | |
| 7 Yiu WH, Wong DW, Chan LY, Leung JC, Chan KW, Lan HY, Lai KN and Tang SC. Tissue kallikrein mediates pro-inflammatory pathways and activation of protease-activated receptor-4 in proximal tubular epithelial cells. PLOS ONE. 2014; 9(2):e88894. | | | | |
| 8 Drews K, Tavernier G, Demeester J, Lehrach H, De Smedt SC, Rejman J and Adjaye J. The cytotoxic and immunogenic hurdles associated with non-viral mRNA-mediated reprogramming of human fibroblasts. BIOMATERIALS. 2012; 33(16):4059-4068. | | | | |
| 9 Botling J, Edlund K, Segersten U, Tahmasebpoor S, Engstrom M, Sundstrom M, Malmstrom PU and Micke P. Impact of thawing on RNA integrity and gene expression analysis in fresh frozen tissue. DIAGN MOL PATHOL. 2009; 18(1):44-52. | | | | |
| 10 Pantou MP, Manginas A, Alivizatos PA and Degiannis D. Connective tissue growth factor (CTGF/CCN2): a protagonist in cardiac allograft vasculopathy development? J Heart Lung Transplant. 2012; 31(8):881-887. | | | | |
